# Assessment of Cardiologists’ Knowledge, Attitudes, and Practices on Calcium Channel Blocker–Induced Gingival Overgrowth

**DOI:** 10.3290/j.ohpd.c_2777

**Published:** 2026-08-13

**Authors:** Zeynep Akgül, Ahmet Çınar

**Affiliations:** a Zeynep Akgül Periodontologist and Asist. Prof. Dr, Department of Periodontology, Faculty of Dentistry, Bolu Abant İzzet Baysal University, Bolu, Türkiye. All contributions to this work are shared equally between the two authors.; b Ahmet Çınar Cardiologist and Medical Doctor, Cardiology Clinic, Samsun City Hospital, Samsun, Türkiye. All contributions to this work are shared equally between the two authors.

**Keywords:** awareness, calcium channel blockers, cardiologists, gingival overgrowth, interdisciplinary communication.

## Abstract

**Purpose:**

Calcium channel blockers (CCBs) are widely used in cardiovascular disease management; however, calcium channel blocker-induced gingival overgrowth remains an underrecognized adverse effect that may impair oral hygiene, exacerbate periodontal inflammation, cause functional and esthetic problems, and potentially affect adherence to cardiovascular therapy. This study aimed to evaluate cardiologists’ knowledge, awareness, and clinical practices regarding DIGO and to explore factors influencing preventive behaviors.

**Materials and Methods:**

This cross-sectional, survey-based study included 139 cardiologists in Türkiye. A structured 25-item questionnaire assessed demographics, oral health awareness, prescribing habits, and clinical practices. Descriptive statistics, chi-square tests, and multivariable binary logistic regression analyses were performed to identify predictors of preventive counseling and oral-focused history taking.

**Results:**

A total of 139 cardiologists participated in the study. Of these, 74.8% were male, and the most common age group was 40–49 years (37.4%). Within the past year, 66.9% had undergone a dental examination, and 47.5% reported a history of periodontal treatment. Knowledge of DIGO pathogenesis was reported by 41.3% of participants, whereas 31.2% stated they had no knowledge. Only 22.3% of cardiologists reported providing preventive oral health advice, while 61.2% referred patients after complications developed. Multivariable analysis revealed that knowledge of DIGO mechanisms was inversely associated with preventive counseling (OR = 0.44, p = 0.004), whereas prior oral health training showed a non-significant but clinically relevant positive trend (OR = 2.24, p = 0.061). Similarly, knowledge-related variables were not positively associated with oral-focused anamnesis.

**Conclusion:**

Cardiologists’ awareness of DIGO remains limited, and their preventive practices are inadequate. The findings reveal a critical knowledge–behavior gap, indicating that increased awareness does not necessarily translate into preventive clinical practice. Interventions targeting behavioral change and structured interdisciplinary collaboration are essential to improve early recognition and management of DIGO.

Calcium channel blockers (CCBs) are widely prescribed for the management of cardiovascular diseases, including hypertension, angina pectoris, and arrhythmias.^16^ Despite their well-established clinical efficacy, these agents are associated with a range of systemic and local adverse effects. Among these, drug-induced gingival overgrowth (DIGO) represents a clinically significant yet often underrecognized condition, particularly in relation to dihydropyridine derivatives, with potential implications for patient management.^8,17^

Periodontal diseases are increasingly recognized not only as localized oral inflammatory conditions but also as conditions with systemic inflammatory relevance. Recent evidence has emphasized the role of serum cytokines in periodontal diseases and their potential contribution to systemic inflammatory patterns. In addition, updated bibliometric, conceptual, and causal-inference studies have further highlighted the close relationship between periodontitis and systemic diseases, supporting the view that periodontal disease may represent a systemic condition rather than an isolated oral problem. Therefore, oral and periodontal health awareness among physicians who manage systemic diseases is clinically important.^2,11,29,34^

Drug-induced gingival overgrowth (DIGO) was first described in 1984 following nifedipine use, and similar effects have since been observed with other agents such as amlodipine, verapamil, and diltiazem. Subsequent reports suggest that both dihydropyridine and non-dihydropyridine CCBs may contribute to this condition.^19^ Interaction with environmental factors such as dental plaque accumulation and poor oral hygiene further exacerbates the process, leading to progressive periodontal deterioration, functional impairment and esthetic concerns. From a pathophysiological perspective, CCB-induced gingival overgrowth results from complex interactions among drug-related effects, gingival fibroblast susceptibility, extracellular matrix metabolism, inflammatory pathways, and local environmental factors. CCBs may alter intracellular calcium-dependent cellular functions, influence fibroblast activity, and disturb collagen turnover, leading to an imbalance between extracellular matrix synthesis and degradation. Plaque-induced inflammation and poor oral hygiene may further amplify this process by increasing inflammatory mediator release and connective tissue accumulation. Therefore, DIGO should not be regarded solely as a pharmacological adverse effect, but rather as a multifactorial condition in which drug exposure interacts with periodontal inflammation, oral hygiene status, and individual susceptibility.

The clinical consequences of DIGO extend beyond gingival enlargement itself. Gingival overgrowth may create pseudopockets, increase plaque retention, and make effective oral hygiene more difficult. These changes may aggravate gingival and periodontal inflammation, contribute to bleeding and discomfort, impair mastication and speech in advanced cases, and cause esthetic concerns. In addition, by promoting plaque retention and compromising plaque control, gingival overgrowth may indirectly contribute to an increased risk of caries, particularly in patients with poor oral hygiene or limited access to preventive dental care. Recent pharmacovigilance-based evidence has also emphasized that DIGO remains a relevant and potentially underestimated adverse drug reaction, particularly in relation to widely prescribed drug groups such as calcium channel blockers.^23^

Although its prevalence varies depending on the specific agent and patient characteristics, its clinical consequences—including esthetic impairment, functional limitations, and potential impact on treatment adherence—underscore its relevance in patient management.^26^

The reported prevalence of DIGO varies according to drug type, dosage, duration of use, genetic predisposition, and oral hygiene status. Rates of up to 20% have been reported among nifedipine users, while the prevalence associated with amlodipine ranges between 3% to 10%.^8,13^ These differences are thought to reflect pharmacokinetic variations among agents. However, predicting which patients will develop DIGO remains difficult, as genetic polymorphisms, sex differences, and concomitant periodontal inflammation may significantly influence disease expression.^15^ Most existing literature on DIGO has focused on pathogenesis, prevalence, and dental management. However, limited attention has been paid to the role of prescribing physicians, particularly cardiologists, in recognizing this condition and managing it through interdisciplinary collaboration. Given that CCBs are primarily prescribed and monitored by cardiologists, their awareness and clinical behaviors are critical for early detection and prevention. Therefore, the level of awareness, knowledge, and clinical attitudes of cardiologists—including residents—toward DIGO is of critical importance for effective multidisciplinary patient care. Although DIGO is well recognized in dental and periodontal research, its relevance may not be sufficiently integrated into routine cardiology practice, reflecting a gap in interdisciplinary awareness and communication. Therefore, evaluating not only knowledge levels but also the relationship between awareness and clinical practice is essential.

Physicians’ knowledge is particularly decisive for patient education, recommendations for dental evaluation, and decisions regarding medication adjustment. Moreover, the esthetic and functional problems caused by DIGO can reduce patient satisfaction, compromise adherence to therapy, and occasionally lead to unnecessary invasive dental procedures. In this context, CCB-associated gingival overgrowth should be regarded not merely as a dental issue but as a systemic complication that directly influences cardiovascular pharmacotherapy.

Given the multifactorial nature of DIGO, its prevention and treatment require a standardized interdisciplinary approach rather than isolated management by a single discipline. From a preventive perspective, cardiologists should be able to identify patients at increased risk, provide basic oral health counseling before or during CCB therapy, and refer patients for dental or periodontal evaluation when necessary. From a treatment perspective, coordinated decision-making between cardiologists and dental professionals is essential to balance cardiovascular benefits with oral health outcomes and to determine whether plaque control, periodontal therapy, drug substitution, dose adjustment, or surgical management is most appropriate. Therefore, structured interdisciplinary communication is central to the early recognition, prevention, and management of CCB-induced gingival overgrowth. The primary aim of this study was to evaluate cardiologists’ knowledge, awareness, and clinical practice behaviors regarding calcium channel blocker-induced gingival overgrowth. The secondary aim was to identify factors associated with preventive oral health counseling and oral-focused history taking, and to assess the perceived need for interdisciplinary collaboration in the prevention and management of this condition.

## MATERIALS AND METHODS

### Study Design and Ethics Approval

This cross-sectional, survey-based study was conducted to evaluate the knowledge, attitudes, and behaviors of cardiologists in Turkey regarding gingival overgrowth associated with calcium channel blockers (CCBs) use. The study was performed in accordance with the Declaration of Helsinki and was approved by the Ethics Committee of Bolu Abant Izzet Baysal University for Non-Interventional Studies (Approval No: 2025/199, Date: 06.05.2025). Electronic informed consent was obtained from all participants prior to enrollment.

### Questionnaire Development and Content Data Availability Statement

The datasets generated and analyzed during the current study are not publicly available due to privacy and ethical restrictions but are available from the corresponding author upon reasonable request.

A structured 25-item questionnaire was developed based on the relevant literature in cardiology and periodontology. The questionnaire assessed demographic characteristics, oral hygiene practices, prescribing tendencies for CCBs, knowledge of drug-induced gingival overgrowth, and clinical management behaviors. Questions regarding participants’ own dental examination history, previous periodontal treatment, self-perceived oral health status and prior oral health education were included to explore whether personal oral/periodontal health experience might be related to awareness and preventive clinical behaviors toward CCB-induced gingival overgrowth.

### Participants and Data Collection

The survey was distributed to physicians via e-mail through the Turkish Society of Cardiology and was additionally shared by the investigators through social-media platforms. Eligible participants included cardiologists working in university hospitals, state hospitals, and private hospitals or clinics. Retired physicians, those working in non-cardiology specialties, and incomplete responses were excluded. Respondents were informed about the purpose and confidentiality of the study and voluntarily completed the survey by accessing the secure link provided in the invitation. No identifying information or personal data were collected.

### Sample Size Calculation

The minimum required sample size was determined using a power analysis for chi-square tests. Based on a medium effect size (w = 0.30) as defined by Cohen 1988, with 4 degrees of freedom (df = 4) and a significance level of α = 0.05, calculations performed using G*Power 3.1.9.6 indicated that at least 120 participants were required to achieve 80% statistical power (1-β = 0.80).

### Statistical Analysis

Data were analyzed using JASP version 0.95 (University of Amsterdam, The Netherlands). Categorical variables were presented as frequencies and percentages. Chi-squared and Fisher’s exact tests were used for group comparisons.

To identify independent predictors of preventive clinical behaviors, multivariable binary logistic regression models were constructed. Two separate models were developed:

Predicting preventive oral health counseling
Predicting oral-focused history taking

Model fit was evaluated using likelihood ratio tests, AIC, BIC, and pseudo-R² indices (McFadden, Nagelkerke, Cox & Snell, and Tjur). Odds ratios (ORs) with 95% confidence intervals (CIs) were reported. A p-value of <0.05 was considered statistically significant.

## RESULTS

### Data Availability Statement

The datasets generated and analyzed during the current study are not publicly available due to privacy and ethical restrictions but are available from the corresponding author upon reasonable request.

### Participant Characteristics

A total of 139 cardiologists participated in the study. The majority were male (74.8%), and the largest age group was 40–49 years (37.4%), followed by 30–39 years (32.4%) and 20–30 years (19.4%). Regarding professional experience, the most common category was 11–15 years (22.3%), while 20.1% of participants identified themselves as research assistants without specifying seniority. The most frequent academic title was Specialist (30.9%), followed by Research Assistant (27.3%) and Associate Professor (20.1%). In terms of workplace, 46.0% worked in the Ministry of Health, 40.3% in universities, and 13.7% in the private sector (Table 1).

**Table 1:** Sociodemographic and professional characteristics of participants

Variable	Category	N	%
Gender	Male	104	74.8
	Female	35	25.2
Age in years	20-30	27	19.4
	30-39	45	32.4
	40-49	52	37.4
	50-59	10	7.2
	≥60	5	3.6
Years in profession	1 to 5	18	12.9
	11 to 15	31	22.3
	16 to 20	21	15.1
	6 to 10	20	14.4
	>20	21	15.1
	Resident	28	20.1
Academic title	Resident	38	27.3
	Specialist	43	30.9
	Assistant Professor	10	7.2
	Associate Professor	28	20.1
	Professor Dr.	20	14.4
Working sector	Ministry of Health	64	46.0
	Private sector	19	13.7
	University	56	40.3
N=number of participants, %=percentage.			

### Oral and Periodontal Health Characteristics

Participants’ oral and periodontal health characteristics were evaluated to describe their personal experience with oral health care, which may influence awareness and preventive practices related to CCB-induced gingival overgrowth. Within this context, 66.9% of participants reported having undergone a dental examination in the past year, whereas only 0.7% reported never having had an examination. Nearly half of the participants (47.5%) had previously received periodontal treatment, while 52.5% had not. In the subjective assessment of oral health, more than half (54.0%) rated their oral health as “good.” Furthermore, 41.7% of participants reported having received prior oral health education, whereas 58.3% had not (Table 2).

**Table 2:** Self-reported oral and periodontal health characteristics of participants

Variable	Category	N	%
Last dental examination	≤1 year	93	66.9
	1–2 years	27	19.4
	>2 years	18	12.9
	Never	1	0.7
Previous periodontal treatment	Yes	66	47.5
	No	73	52.5
Self-assessment of oral health	Very good	6	4.3
	Good	75	54.0
	Fair	47	33.8
	Poor	11	7.9
Previous oral health education	Yes	58	41.7
	No	81	58.3
N=number of participants, %=percentage.			

### Attitudes and Practices Toward CCB-Induced Gingival Overgrowth

Regarding attitudes and practices toward patients prescribed calcium channel blockers (CCBs), only 22.3% of participants reported providing preventive oral health advice to patients using CCBs. In contrast, 61.2% referred patients who developed drug-induced gingival overgrowth to a dentist. The rate of inquiring about oral health and gingival overgrowth during anamnesis was lower, with only 32.4% reporting this practice. The majority of participants (94.2%) prescribed dihydropyridine-type CCBs, while 5.8% prescribed non-dihydropyridines (Table 3).

**Table 3:** Attitudes, practices, and prescription patterns regarding calcium channel blocker-induced gingival overgrowth

Variable	Category	N	%
Do you provide oral health advice to patients prescribed CCBs?	Yes	31	22.3
	No	108	77.7
Do you refer patients with CCB-induced gingival overgrowth to a dentist?	Yes	85	61.2
	No	54	38.8
Do you take anamnesis regarding oral health and gingival overgrowth in patients prescribed CCBs?	Yes No	45 94	32.4 67.6
Most frequently prescribed CCB type?	Dihydropyridines	131	94.2
	Non-dihydropyridines	8	5.8
Perceived frequency of CCB-induced gingival overgrowth	Very rare	40	28.8
	Rare	95	68.3
	Common	4	2.9
CCB subgroup most frequently associated with gingival overgrowth	Dihydropyridines	118	84.9
	Non-dihydropyridines	21	15.1
Dihydropyridines most associated with gingival overgrowth	Nifedipine	27	19.42
	Felodipine	4	2.88
	Lercanidipine	19	13.67
	Lacidipine	1	0.72
	Amlodipine	96	69.06
	Benidipine	12	8.63
Non-dihydropyridines most associated with gingival overgrowth	Verapamil	15	71.43
	Diltiazem	9	42.86
CCB: calcium channel blocker; N: number of participants; %: percentage.			

### Prescription Habits and Awareness of Gingival Overgrowth

With respect to prescribing habits and awareness, 94.2% of cardiologists reported prescribing dihydropyridine drugs. Most participants considered gingival overgrowth to occur rarely (68.3%) or very rarely (28.8%), while only 2.9% viewed it as common. Among specific agents, amlodipine was most frequently associated with gingival overgrowth (69.1%), followed by nifedipine (19.4%) and lercanidipine (13.7%). Less frequently reported agents included benidipine (8.6%), felodipine (2.9%), and lacidipine (0.7%). In the non-dihydropyridine group, gingival overgrowth was most commonly observed with verapamil (71.4%) and diltiazem (42.9%) (Table 4).

**Table 4:** Knowledge and approaches regarding gingival overgrowth induced by calcium channel blockers

Frequencies of Participants’ Knowledge Regarding CCB Medications			
Variable	Level	N	%
Are you aware of the mechanisms by which calcium channel blockers (CCBs) cause gingival overgrowth?	Yes	57	41.3
	Partially	38	27.5
	No	43	31.2
How would you evaluate the frequency of gingival overgrowth caused by CCBs?	Very rare	40	28.8
	Rare	95	68.3
	Common	4	2.9
What is your treatment approach for patients who develop gingival overgrowth during CCB therapy?	Dose reduction or switching the drug	116	85.3
	Emphasis on oral hygiene, dental cleaning, and root planing	12	8.8
	Gingival surgery	2	1.5
	No intervention needed	6	4.4
In which patients is the risk of gingival overgrowth associated with CCB use higher?	Poor oral hygiene	38	27.3
	Diabetic patients	5	3.6
	Young patients	7	5
	Pregnant women	15	10.8
	Elderly patients	38	25.9
	None	36	27.3
Do you regularly receive training on new research and treatment protocols related to CCBs?	Yes	20	14.4
	Maybe	55	39.6
	No	64	46
Do you think a multidisciplinary approach is beneficial for CCB-induced gingival overgrowth?	Yes	131	94.2
	No	8	5.8
	Unsure	0	0
What is the biggest barrier you face when making clinical decisions regarding CCBs and gingival overgrowth?	Patient’s poor oral hygiene	24	17.52
	Patients’ reluctance to change dose or medication	15	10.95
	Need for surgical intervention	2	1.46
	Balancing the effects of CCBs	9	6.57
	Lack of multidisciplinary collaboration	52	37.96
	Complexity of treatment options	6	4.38
	Insufficient knowledge	29	21.17
Do you think further research is needed on CCB-related gingival overgrowth?	No	12	8.6
	Yes	101	72.7
	Unsure	26	18.7
Which common side effects of CCBs, other than gingival overgrowth, are you aware of?	Peripheral edema	132	94.96
	Constipation	89	64.03
	Dizziness	34	24.46
	Skin rashes	15	10.79
	Dry mouth	20	14.39
	Allergic reaction	28	20.14
	Hypotension	60	43.17
CCB: calcium channel blocker; N: number of participants; %: percentage			

### Knowledge, Management Strategies, and Training

Regarding knowledge about the mechanisms of CCB-induced gingival overgrowth, 41.3% of participants stated that they were knowledgeable, 27.5% were partially aware, and 31.2% were unaware. The most frequently reported management strategy was dose reduction or medication change (85.3%), followed by oral hygiene instruction and scaling (8.8%), whereas surgical intervention was rarely preferred (1.5%). Participants most frequently identified patients with poor oral hygiene (27.3%) and elderly individuals (25.9%) as high-risk groups. Only 14.4% of cardiologists reported receiving regular training on CCB treatment protocols, and 94.2% emphasized the importance of a multidisciplinary approach. The most frequently recognized side effects other than gingival overgrowth were peripheral edema (95.0%) and constipation (64.0%) (Table 5). Although oral hygiene instruction was recognized by some participants as part of the management of CCB-induced gingival overgrowth, preventive counseling and oral-focused history taking were not routinely reported.

**Table 5:** Association between academic title and receiving oral health education

Cross-Tabulation of Professional Title and Previous Oral Health Education				
Professional Title			Previous Oral Health Education	
			No	Yes
	Value	Df	p	
Research assistant		Observed	15.00	23.00
		Expected	22.14	15.86
		% within row	39.474	60.526
Specialist		Observed	25.00	18.00
		Expected	25.06	17.94
		% within row	58.14	41.86
Assistant professor		Observed	6.00	4.00
		Expected	5.83	4.17
		% within row	60.00	40.00
Associate professor		Observed	19.00	9.00
		Expected	16.32	11.68
		% within row	67.86	32.14
Professor		Observed	16.00	4.00
		Expected	11.65	8.34
		% within row	80.00	20.00
% within row indicates the percentage of participants in each education category within the same professional title. Expected values are calculated based on marginal totals under the assumption of independence.				
Chi-squared test results for professional title and previous oral health education				
Χ²	10.05	4	0.007	
N	139			
The Pearson chi-squared test indicates a statistically significant association between professional title and previous oral health education (χ^2^ = 10.05, p = 0.007).				

### Associations Between Variables

A significant association was found between oral health training and academic title. Training was most frequent among research assistants (60.5%) and least common among professors (20.0%), indicating a decline with higher academic seniority.

Additionally, a significant difference was observed between work sector and the type of CCB most often associated with gingival overgrowth (χ^2^ = 10.48, df = 4, p = 0.033). Physicians working in the Ministry of Health (92.2%) and private sector (94.7%) more frequently reported dihydropyridines, whereas university physicians more often reported non-dihydropyridines (26.8%) (Table 6).

**Table 6:** Association between working sector and type of CCB-induced gingival overgrowth encountered

Cross-tabulation of sector and type of CCB causing gingival overgrowth								
Working sector				Most common CCB causing gingival overgrowth?				Total
				Dihydropyridine		Non-dihydropyridine		
			Value		df		p	
Ministry of Health		Observed		59.00		5.00		64.00
		Expected		54.33		9.67		64.00
		% within row		92.19		7.81		100
Private sector		Observed		18.00		1.00		19.00
		Expected		16.13		2.87		19.00
		% within row		94.74		5.26		100
University		Observed		41.00		15.00		56.00
		Expected		47.54		8.46		56.00
		% within row		73.21		26.79		100
Total		Observed		118.00		21.00		139.00
		Expected		118.00		21.00		139.00
		% within row		84.89		15.11		100
CCB: calcium channel blocker; % within row indicates the proportion of participants in each CCB category within the same sector. Expected values are calculated based on marginal totals under the assumption of independence.								
Chi-squared test results for sector and type of CCB causing gingival overgrowth								
Χ^2^			10.48		4		0.033	
N			139					
CCB: calcium channel blocker; the Pearson chi-squared test indicates a statistically significant association between the participants’ sector and the type of calcium channel blocker (CCB) associated with gingival overgrowth (χ^2^ = 10.48, p = 0.033).								

### Multivariable Analysis of Predictors of Preventive Counseling and Oral-Focused History Taking

Multivariable binary logistic regression analyses were performed to identify independent predictors of preventive counseling and oral-focused history taking behaviors among cardiologists. In the model predicting preventive oral health counseling, the overall model demonstrated a significantly better fit compared to the null model (Δχ^2^ = 12.49, p = .002) (Table 7). The explanatory power of the model was modest (Nagelkerke R^2^ = 0.131). Awareness of the mechanisms linking calcium channel blockers to gingival overgrowth was significantly associated with preventive counseling in an inverse direction (OR = 0.44, 95% CI: 0.26–0.77, p = 0.004). Prior oral health training showed a positive but non-significant association with preventive counseling (OR = 2.24, 95% CI: 0.96–5.20, p = 0.061) (Table 8). In the model predicting oral-focused history taking, the model also showed a significantly improved fit compared to the null model (Δχ^2^ = 8.34, p = .015) (Table 9), although the explanatory power remained limited (Nagelkerke R^2^ = 0.081). Neither regular training nor perceived frequency of gingival overgrowth showed a statistically significant positive association with history taking behavior. Both variables demonstrated borderline significance with inverse relationships (OR = 0.59, p=0.059 and OR = 0.46, p=0.050, respectively) (Table 10). Overall, the findings indicate that knowledge- and training-related variables were not positively associated with preventive clinical behaviors.

**Table 7:** Model fit indices for the binary logistic regression predicting preventive oral health counseling

Model	Deviance	AIC	BIC	Δχ²	p	McFadden R^2^	Nagelkerke R^2^
Null model (M_0_)	147.5	149.536	152.471	—	—	0.000	0.000
Final model (M_1_)	135.0	141.046	149.850	12.49	.002	0.085	0.131


**Table 8:** Multivariable logistic regression analysis predicting preventive oral health counseling

Variable	OR	95% CI	p
Oral health training	2.24	0.96–5.20	0.061
Awareness of DIGO mechanisms	0.44	0.26–0.77	0.004


**Table 9:** Model fit indices for the binary logistic regression predicting oral-focused history taking

Model	Deviance	AIC	BIC	Δχ^2^	p	McFadden R^2^	Nagelkerke R^2^
Null Model (M₀)	175.0	177.045	179.979	—	—	0.000	0.000
Final Model (M₁)	166.7	172.702	181.505	8.34	0.015	0.048	0.081


**Table 10:** Multivariable logistic regression analysis predicting oral-focused history taking

Variable	OR	95% CI	p
Regular CCB-related training	0.59	0.34–1.02	0.059
Perceived DIGO frequency	0.46	0.22–1.00	0.050


### DISCUSSION

Calcium channel blockers (CCBs) are widely used agents in the treatment of cardiovascular diseases, and drug-induced gingival overgrowth (DIGO) represents a clinically relevant yet frequently underrecognized adverse effect with potential implications for patient management.^7,10^ Although previous studies have extensively investigated the prevalence, risk factors, and pathogenesis of DIGO, research focusing on physicians’ awareness and clinical practices remains limited.^6,8,24^

This study provides novel and clinically relevant insights into cardiologists’ knowledge and clinical practices regarding DIGO and, more importantly, reveals a consistent knowledge–behavior gap. Although this study was conducted in a specific national context, the observed knowledge–behavior gap likely reflects a broader challenge in interdisciplinary clinical practice and may be relevant across different healthcare systems.^31^

In our study, only 41.3% of participants reported having knowledge about the mechanisms of DIGO, while a considerable proportion demonstrated partial or insufficient awareness. This finding indicates a substantial gap in awareness, which may contribute to variability in clinical decision-making and may hinder early recognition and timely management of DIGO. Similar studies have reported lower levels of awareness. Ugale et al^32^ reported that only 26% of medical doctors could identify CCBs among the drugs associated with gingival overgrowth, while Özdemir and Uzunkaya^22^ found that only 19% could do so. Furthermore, Sharma et al^27^ reported low awareness of drug-related side effects, and Sridharan et al^28^ emphasized that oral side effects associated with CCBs are frequently underreported. Although most previous studies have focused primarily on awareness levels, recent evidence has begun to examine whether this awareness translates into clinical practice.

A recent cross-sectional study from Iraq by Ali et al^3^ provides an important comparison with the present findings. In that study, 331 physicians working in primary healthcare institutions were surveyed regarding their awareness and clinical practice related to CCB-induced gingival enlargement. The authors reported that 58% of physicians recognized CCBs as a possible cause of gingival enlargement; however, only 33.5% informed patients about this risk, and 57.7% did not regularly refer patients for gingival assessment. These findings are consistent with the present study, in which 41.3% of cardiologists reported knowledge of DIGO mechanisms, but only 22.3% provided preventive oral health advice and 32.4% obtained oral-focused anamnesis. However, our study differs from Ali et al. by focusing specifically on cardiologists, who are directly responsible for prescribing and monitoring CCB therapy, and by using multivariable regression models to evaluate predictors of preventive counseling and oral-focused history taking. Together, these studies suggest that the gap between awareness and preventive clinical behavior is not limited to a single healthcare system but may represent a broader challenge in integrating oral health considerations into medical practice.

One of the most striking aspects of our findings is the differences in education and awareness levels according to academic title, sector, and age. Research assistants reported receiving significantly more oral health education compared with professors. On the other hand, Alim et al^4^ reported that there was no correlation between the knowledge of cardiologists and cardiovascular surgeons on periodontal disease and their years of experience or professional seniority. These differences may reflect variations in clinical workload, access to continuing education, and exposure to interdisciplinary collaboration.

Physicians working in the Ministry of Health and the private sector reported encountering dihydropyridine-related DIGO more frequently than those working in university hospitals. The higher incidence of non-dihydropyridine-related cases in university hospitals can be explained by the more widespread use of alternative drugs and the higher prevalence of complex cardiac cases in these centers. When age distribution was examined, it was found that participation in training was quite low among younger physicians, but this rate increased to 26.7% in the 50–59 age group. These findings show that education and awareness levels vary according to professional seniority, work environment, and age. The sector-related difference observed in our study may reflect variations in prescribing patterns, patient profiles, clinical exposure and access to structured interdisciplinary communication. Therefore, standardized training programs involving both cardiologists and dental professionals are needed to improve risk recognition, preventive counseling, oral-focused history taking and referral practices across different healthcare sectors. Such training may be particularly valuable in private-sector settings, where interdisciplinary referral pathways and continuing education opportunities may be less formally structured.

Amlodipine is a long-acting calcium channel blocker belonging to the dihydropyridine group and was first associated with gingival overgrowth by Seymour et al^25^ in 1994. Most of our participants reported encountering amlodipine-related gingival overgrowth most frequently in clinical practice. The literature also reports that gingival overgrowth is most commonly associated with dihydropyridines, with reported rates ranging from 20% to 83% for nifedipine and initially 1–3% for amlodipine.^14^ More recent studies have reported higher rates, such as 31.4% by Gopal et al^9^ and up to 76% by Jayanthi et al.^12^ Although amlodipine is not the biologically highest-risk agent, its widespread prescription in clinical practice explains why physicians encounter more cases of gingival overgrowth with this drug. This may also contribute to the perception that DIGO is drug-specific rather than patient- or risk-dependent. According to our findings, non-dihydropyridines are prescribed less frequently, with verapamil and diltiazem being the most prominent among them.

Gingival inflammation is considered the most important determinant of the prognosis of drug-induced gingival overgrowth. In addition, age, sex, systemic conditions, and genetic predisposition are other factors affecting prognosis.^6,24^ In our study, participants identified patients with poor oral hygiene (27.3%) and elderly individuals (25.9%) as the highest risk groups. Meanwhile, 27.3% stated that no specific risk group existed. This finding suggests that cardiologists may not be sufficiently aware of oral hygiene habits, which are among the most important factors affecting prognosis. This is particularly important, as oral hygiene has been consistently identified as a key determinant of DIGO severity in previous studies.^6,24^

In the treatment of DIGO, medication change and dose adjustment are generally considered together with oral hygiene education, professional dental scaling, root planing, and, when necessary, periodontal surgery.^5,30^ Importantly, several reports have shown that DIGO may be managed without medication change in selected cases, particularly through effective plaque control, reduction of gingival inflammation, and periodontal treatment.^6,21^ In our study, 85.3% of participants preferred dose reduction or medication change, while only 8.8% considered periodontal treatment, 1.5% surgical intervention, and 4.4% no intervention. In addition, 37.9% of participants reported lack of interdisciplinary collaboration as the most significant challenge in clinical decision-making, while 21.2% cited the complexity of treatment options. This discrepancy suggests that cardiologists may tend to prioritize pharmacological modification over dental and periodontal management. Therefore, structured interdisciplinary guidelines are needed to support timely dental treatment and periodontal inflammation control before medication change is considered, when clinically appropriate.

Gingival overgrowth also requires careful differential diagnosis. Although CCB therapy is an important drug-related cause, gingival enlargement should not automatically be attributed to medication use alone. Biofilm-associated gingival inflammation, hormonal influences, vitamin deficiencies, hematological disorders such as leukemia, granulomatous diseases such as sarcoidosis, and other drug-related causes should also be considered.^1^ Local inflammation and plaque retention may coexist with DIGO and aggravate tissue enlargement. Therefore, timely dental/periodontal evaluation is essential to distinguish CCB-induced gingival overgrowth from other local or systemic causes and to guide appropriate management.

Current cardiology and hypertension guidelines emphasize the cardiovascular indications, drug selection, blood pressure targets, and systemic adverse effects of calcium channel blockers; however, specific recommendations regarding the prevention, prevalence, dental/periodontal referral, and treatment of CCB-induced gingival overgrowth are limited and are not explicitly incorporated into routine cardiology guidance. This gap may be clinically important. Although some cardiologists may be aware that oral hygiene instruction and periodontal care are relevant in DIGO management, the absence of standardized cardiology-based guidance may limit the translation of this awareness into routine preventive counseling, oral-focused history taking, and timely interdisciplinary referral.^18,20^

An important finding of the present study was that only 2.9% of cardiologists perceived CCB-induced gingival overgrowth as common, whereas most participants considered it rare or very rare. This low perceived frequency may not necessarily reflect the true clinical occurrence of DIGO but may instead indicate underrecognition in routine cardiovascular practice. Because gingival overgrowth may develop gradually and remain mild or asymptomatic in its early stages, it may not be reported by patients unless it causes discomfort, esthetic concern, bleeding, or functional limitation. Moreover, cardiologists may primarily focus on systemic adverse effects of CCBs and may not routinely inspect oral manifestations during follow-up visits. In the present questionnaire, we evaluated whether participants asked about oral health and gingival overgrowth during anamnesis; however, we did not specifically distinguish between regular clinical inspection of oral manifestations and recognition only after patient-reported complaints. The predominance of reactive referral patterns rather than preventive strategies indicates a clinically relevant gap in early intervention. The fact that most cardiologists refer patients only after complications develop suggests that DIGO is not integrated into routine clinical assessment. This reactive approach may lead to delayed diagnosis, increased need for invasive periodontal treatment, potential compromise in patient adherence to cardiovascular therapy, and may ultimately increase healthcare burden due to preventable complications. Similar patterns of delayed referral and limited preventive practice have been reported in different populations.^22,32,33^

Contrary to conventional assumptions, increased knowledge and awareness of DIGO were not associated with improved preventive clinical behaviors. In some instances, higher awareness was even associated with a lower likelihood of providing preventive counseling or obtaining oral health-related anamnesis. These findings highlight a critical and clinically relevant knowledge–behavior gap, indicating that awareness alone is insufficient to drive clinical practice. Although previous studies have primarily focused on awareness levels, few have explored this discrepancy between knowledge and clinical behavior, highlighting the novelty of the present findings. This finding challenges the traditional assumption that increasing awareness alone is sufficient to improve clinical practice.

One possible explanation for this disconnect is role perception. Cardiologists may recognize DIGO as a drug-related adverse effect but still perceive its management as belonging primarily to dental professionals. This reflects a potential fragmentation in responsibility between medical and dental disciplines. Additionally, clinical prioritization, time constraints, and the lack of structured interdisciplinary communication pathways may further limit the translation of knowledge into practice.

Although prior oral health training showed a positive trend, its lack of statistical significance suggests that knowledge-based interventions alone may be insufficient. The modest explanatory power of the regression models further supports this interpretation, indicating that clinical behavior is influenced by broader systemic and organizational factors beyond individual knowledge and suggesting that unmeasured behavioral and systemic factors may play a substantial role. These findings, derived from multivariable regression models, further emphasize that knowledge alone is not an independent determinant of preventive clinical behavior.

From a broader perspective, these findings align with behavior change theories, such as the COM-B model (Capability, Opportunity, Motivation–Behavior), which emphasize that knowledge alone is insufficient to drive behavior and that structural and contextual factors play a critical role. Therefore, improving DIGO management requires not only increasing awareness but also integrating oral health into cardiology training, developing structured referral pathways, and embedding preventive practices into routine care. Future studies should evaluate structured interventions for interdisciplinary DIGO management, including standardized training programs jointly developed by cardiologists and dental professionals, referral algorithms, and practical clinical guidance for risk assessment, preventive counseling, oral-focused history taking, and periodontal referral. In addition, cardiologists’ perceived usefulness, acceptability, and feasibility of such interdisciplinary training should be assessed, as these factors may determine whether educational interventions can be translated into routine clinical practice.

From a clinical perspective, the findings of this study suggest several practical recommendations. Cardiologists prescribing calcium channel blockers should inform patients about the possibility of gingival overgrowth, especially in patients with poor oral hygiene, existing periodontal inflammation, or long-term CCB use. Oral health-related questions should be incorporated into routine anamnesis, and patients should be encouraged to undergo regular dental or periodontal evaluation. When gingival enlargement is suspected, timely referral to a dentist or periodontist is recommended before considering medication change, provided that the patient’s cardiovascular status allows coordinated decision-making. Therefore, effective DIGO management should rely on early recognition, preventive counseling, plaque control, periodontal treatment, and structured interdisciplinary communication between cardiologists and dental professionals.

This study has several limitations. Its cross-sectional design limits causal interpretation, and the use of self-reported data may introduce reporting bias. Furthermore, the absence of clinical examination refers to the inability to objectively verify the presence or severity of gingival overgrowth, which may have affected the accuracy and clinical interpretability of the findings. In addition, the questionnaire did not specifically determine whether cardiologists routinely inspected oral manifestations during clinical follow-up or recognized gingival overgrowth only after patient-reported complaints.

## CONCLUSION

This study demonstrates that cardiologists’ knowledge and awareness of drug-induced gingival overgrowth (DIGO) do not consistently translate into preventive clinical practices, revealing a clinically relevant knowledge–behavior gap. Despite moderate levels of awareness, preventive counseling remains limited and clinical management is predominantly reactive, with referrals often occurring only after complications develop. This pattern suggests that DIGO is not adequately integrated into routine clinical assessment. These findings indicate that awareness-based educational approaches alone may be insufficient to influence clinical behavior. Instead, behavioral, organizational, and interdisciplinary factors play a crucial role in shaping clinical practice. Therefore, improving DIGO management requires a comprehensive and standardized interdisciplinary approach that includes integrating oral health into cardiology training, strengthening structured communication between cardiologists and dental professionals, incorporating preventive oral health assessment into routine cardiovascular care and coordinating treatment decisions when gingival overgrowth develops. Future studies should focus on developing and evaluating interventions that target behavioral change and interdisciplinary integration.
